# Clinical Outcomes of Intra‐Abdominal Candidiasis by Initial Antifungal Therapy

**DOI:** 10.1111/myc.70147

**Published:** 2026-03-03

**Authors:** M. Albanell‐Fernández, A. Vergara, F. Marco, S. Herrera, M. Tuset, A. Soriano, M. Bodro

**Affiliations:** ^1^ Pharmacy Service, Division of Medicines, Hospital Clinic of Barcelona Universitat de Barcelona Barcelona Spain; ^2^ Department of Physiological Science, School of Medicine Universitat de Barcelona, L'hospitalet de Llobregat Barcelona Spain; ^3^ Department of Clinical Microbiology, Hospital Clinic‐CDB Universitat de Barcelona Barcelona Spain; ^4^ Institute of Global Health (ISGlobal) Barcelona Spain; ^5^ CIBER Enfermedades Infecciosas (CIBERINFEC) Instituto Salud Carlos III Madrid Spain; ^6^ Department of Infectious Diseases, Hospital Clinic of Barcelona Universitat de Barcelona Barcelona Spain; ^7^ Institut D'investigació Biomèdica August Pi I Sunyer (IDIBAPS) Barcelona Spain; ^8^ Faculty of Medicine University of Barcelona Barcelona Spain

**Keywords:** azoles, candidemia, echinocandins, intra‐abdominal candidiasis, mortality, persistence

## Abstract

**Background:**

Intra‐abdominal candidiasis (IAC) is a severe and heterogeneous infection associated with significant morbidity and mortality.

**Objectives:**

To evaluate outcomes of IAC according to initial antifungal therapy and to identify predictors of persistent infection and 30‐day mortality.

**Methods:**

A retrospective non‐randomised single‐centre study (January 2020–February 2025) evaluated adult IAC according to the initial antifungal (azoles vs. echinocandins), assessing demographics, type, location, and persistent positive cultures, re‐intervention, antifungal reintroduction, and 3‐ and 90‐day mortality. Multivariate logistic regression identified predictors of persistent cultures and 30‐day mortality, and inverse probability of treatment weighting (IPTW) addressed confounding in mortality analyses.

**Results:**

Among 154 patients (median age 64.5 years, 66.9% male), 102 received azoles and 52 echinocandins, with greater severity in the latter group. Persistent positive cultures occurred in 51.7% (46/89), without difference between groups. Factors associated with persistent positive cultures included concomitant candidemia, septic shock, previous antibiotic usage, and peritonitis. The 30‐day mortality was 28.1% (higher with echinocandins, 38.5% vs. 22.8% in azole group, *p* = 0.041). Multivariate logistic regression and IPTW indicated that the initial antifungal therapy did not affect 30‐day mortality, while septic shock (OR:2.2, 95% CI:1.0–4.9; *p* = 0.047) and age ≥ 60 years (OR:2.6, 95% CI:1.1–6.3; *p* = 0.032) were significantly associated with it.

**Conclusions:**

IAC remains a complex infection with substantial morbidity and mortality. Echinocandins are preferred in critically ill patients, but mortality did not differ significantly between initial echinocandin and azole treatment. Persistent positive cultures were linked to severe presentation, including peritonitis, candidemia, and septic shock, which required more frequent re‐intervention, and consequently carried higher mortality.

## 1 Introduction

Intra‐abdominal candidiasis (IAC) is diagnosed by isolating *Candida* spp. from peritoneal fluid in patients with clinical signs of infection [1, 2]. Its diagnosis is challenging due to nonspecific symptoms and the complexity of interpreting the pathogenic significance of the isolation of fungi from intra‐abdominal samples [3, 4]. Complicated IAC requires adequate source control and adequate antifungal therapy [5]. The isolation of *Candida* spp. is common in secondary peritonitis, particularly in upper gastrointestinal perforation and among patients with necrotizing pancreatitis. These cases often require multiple surgical interventions and frequently occur after exposure to broad‐spectrum antibiotics [2, 3, 6, 7, 8]. These conditions likely contribute to the high reported mortality of those cases, ranging from 20% to 41% [5, 9, 10, 11, 12], and reaching up to 52% in ICU patients [13].

Timely and effective treatment is essential to improving clinical outcomes and reducing the high mortality associated with IAC. Although clinical guidelines for the management of IAC are available, there remains a significant need for further evidence to substantiate current therapeutic recommendations [2, 4, 14]. Most guidelines recommend the same antifungal agents used for candidemia, with echinocandins considered the first‐line empiric treatment, particularly in severe infections [14, 15, 16]. The recent 2025 guidelines for the diagnosis and management of candidiasis recommend both echinocandins and fluconazole for the treatment of IAC, as current evidence is largely derived from observational studies, with no randomised head‐to‐head comparisons available [17]. However, there is limited penetration of echinocandins into the peritoneal cavity, raising concerns about the adequacy of current therapeutic approaches in this context [18, 19, 20].

The objective of this study was to evaluate the clinical outcomes of patients diagnosed with IAC based on initial antifungal treatment received, either azoles or echinocandins, and to identify key factors associated with persistent positive cultures and mortality.

## 2 Patients and Methods

### 2.1 Study Design and Definitions

A single‐center, retrospective study was conducted involving adult patients diagnosed with IAC at a tertiary care hospital between January 2020 and February 2025. Patients were identified through review of microbiological cultures yielding *Candida* spp. from intra‐abdominal specimens.

IAC was defined as the presence of clinical evidence of intra‐abdominal infection (elevated inflammatory markers and/or fever with associated inflammation) combined with the isolation of *Candida* spp. from a specimen obtained under sterile conditions, either intraoperatively or from a drain placed within 24 h after surgery. All included IAC episodes required antifungal therapy with either azoles or echinocandins. Patients receiving liposomal amphotericin B as the main treatment were excluded from the analysis.

Patients who received antifungal therapy for more than 3 days were included and classified according to the antifungal administered during the first 5 days of therapy. For patients with multiple IAC episodes, only the first episode was included in the analysis. Antifungal susceptibility data were recorded when available.

### 2.2 Data Collection

The collected variables included age, sex, location at the time of the IAC episode (ICU or general ward), admission service, type of IAC infection (according to the classification proposed by Vergidis et al. [5]), site of origin, and the presence of septic shock (persistent hypotension requiring vasopressor support to maintain a mean arterial pressure ≥ 65 mmHg and a serum lactate level > 2 mmol/L despite adequate fluid resuscitation) at the diagnosis of the IAC. The following predisposing factors for IAC were collected based on previously published literature [3, 5, 12, 13]: parenteral nutrition, immunosuppression condition, corticosteroid therapy, upper gastrointestinal perforation, the use of antibiotics and antifungals within the preceding 4 days, and multifocal *Candida* spp. colonisation, defined as the isolation of *Candida* in cultures obtained from the following sources: respiratory tract secretions, rectal swabs, skin, or urine, without evidence of tissue invasion or systemic infection.

Data on source control (surgical or percutaneous), antifungal therapy administered in the 4 days prior to diagnosis, and the antifungal treatment used specifically for IAC were also collected. Source control was defined as the adequate drainage of infected material combined with surgical correction of the underlying pathology, such as perforation or an anastomotic leak. Active antifungal treatment was defined as: (1) administration of an antifungal agent with documented in vitro activity against the isolated *Candida* spp. (when susceptibility testing was available); or (2) the use of an agent to which the isolated species was not intrinsically resistant. Breakthrough IAC was defined as the diagnosis of IAC in a patient who was under active treatment for at least 3 days before the isolation of the causative pathogen. Early initiation of antifungal therapy was defined as treatment starting within 2 days of the culture collection. The total length of hospital stay and ICU stay was also recorded.

The primary endpoints were persistence of positive cultures from intra‐abdominal samples and 30‐day mortality after finishing the antifungal treatment. Persistence was considered when *Candida* spp. was isolated in a follow‐up culture obtained under the conditions described above, after source control and ≥ 72 h of active antifungal therapy. Mortality at the end of treatment and at 90 days, need for surgical reintervention, duration of antifungal treatment, recurrent IAC, and antifungal reintroduction in the following 90 days after ending treatment were secondary endpoints. Recurrent infection was defined as a culture‐confirmed episode occurring after documented microbiological resolution, evidenced by 2 consecutive negative cultures, or after radiographic resolution of the initial infection.

### 2.3 Microbiological Methods

Clinical samples were inoculated onto blood agar, chocolate agar, and Schaedler agar plates and incubated at 37°C for 48 h. In addition, an enrichment broth was included and incubated for 5 days. Some samples were also inoculated into Bactec bottles (bioMérieux) and incubated for up to 5 days. When yeast growth was observed, the isolates were subcultured onto Sabouraud agar for antifungal susceptibility testing. Species identification was performed by MALDI‐TOF mass spectrometry (Bruker).

Concomitant candidemia was diagnosed using blood cultures processed in the Bactec system (bioMérieux) according to standard clinical microbiology procedures.

Antifungal susceptibility testing (AFST) was carried out using the commercial Sensititre YeastOne (Thermo Fisher Scientific) system, and MIC results were interpreted based on the EUCAST species‐specific breakpoints (V11.0).

### 2.4 Statistical Analysis

Descriptive statistics were used to summarise the cohort according to the antifungal therapy (azoles or echinocandins) received during the first 5 days of treatment. Associations between categorical variables were analysed using Pearson's chi‐square test or Fisher's exact test, as appropriate, while continuous variables with a non‐normal distribution were analysed using the Mann–Whitney test. Variables with a *p* < 0.1 in univariate analyses were included in a multivariate logistic regression model to identify independent risk factors for persistent positive cultures and 30‐day mortality. Model performance and goodness‐of‐fit for the logistic regression models were evaluated using the area under the receiver operating characteristic curve and the Hosmer‐Lemeshow test, respectively.

To address confounding by indication related to the choice of initial antifungal therapy in the analysis of 30‐day mortality, we applied inverse probability of treatment weighting (IPTW) based on propensity scores. The propensity score for receiving echinocandin therapy was estimated through a logistic regression model including baseline covariates. Stabilised weights for the average treatment effect on the treated (ATET) were then computed. Covariate balance before and after weighting was assessed using standardised mean differences, with values *p* < 0.1 considered indicative of adequate balance. The effect of initial antifungal therapy on 30‐day mortality was subsequently estimated in the weighted pseudo‐population using an IPTW estimator of the ATET. A *p* < 0.05 was considered statistically significant. Analyses were performed using STATA/SE version 17.0 (STATA, College Station, TX).

### 2.5 Ethics

The study was approved by the Ethics Committee of the Hospital Clinic de Barcelona (Reference: HCB/2025/0350). The need for informed consent was waived due to the noninterventional, retrospective characteristics of the study.

## 3 Results

### 3.1 Clinical Characteristics of IAC

A total of 206 episodes of IAC were identified between January 2020 and February 2025. Fifty‐two cases were excluded for the following reasons: (1) the isolates were considered a colonisation according to an infectious diseases specialist (*n* = 30); (2) the patient died within 7 days of a positive culture without receiving treatment (*n* = 12); (3) the patient received < 3 days of antifungal therapy due to early death (*n* = 9); and (4) the patient was treated with liposomal amphotericin B (*n* = 1). The final cohort included 154 patients, 66.9% were male, with a median [IQR] age of 64.5 [54.5–75.8] years. Ninety‐three patients (60.4%) developed IAC during ICU admission. The most common types of IAC were intra‐abdominal abscesses (47.4%, 73/154) and peritonitis (46.8%, 72/154). All patients underwent source control, 73.4% (113/154) by a surgical procedure and 26.6% (41/154) by percutaneous drainage. Concomitant candidemia occurred in 12.3% (19/154) of cases. Overall, 38.3% (59/154) received active antifungal therapy within 2 days of culture collection.

One hundred two patients were treated with azoles during the first 5 days after diagnosis, and 52 with echinocandins (Table 1). No statistically significant differences were found between the groups in reference to the site of infection origin; however, pancreatic location was numerically more frequent in the group primarily treated with echinocandins (38.5% vs. 24.5%). Patients treated with echinocandins had a significantly higher proportion of concomitant candidemia (26.9% vs. 4.9%, *p* < 0.001), ICU admissions (75% vs. 47.1%, *p* = 0.009), septic shock (51.9% vs. 27.5%, *p* = 0.003), and breakthrough IAC (21.2% vs. 4.9%, *p* = 0.002) compared with those initially treated with azoles. The median [IQR] hospital and ICU lengths of stay were longer in the echinocandin group: 37 [23–61] vs. 28.5 [17–52] days (*p* = 0.042) for hospital stay, and 16 [10–28] vs. 6.5 [3–14] days (*p* < 0.001) for ICU stay.

**TABLE 1:** Clinical characteristics of patients with intra‐abdominal candidiasis according to the antifungal treatment within the first five days.

	Azole treatment (*N* = 102)	Echinocandin treatment (*N* = 52)	*p* ^a^
Age (years), median [IQR]	63.8 [54.5–75.8]	66.6 [54.4–75.8]	0.669
Male Sex	69 (67.7)	34 (65.4)	0.778
Admission service			
Hepatology	12 (11.8)	7 (13.5)	0.763
Digestive surgery	70 (68.6)	29 (55.7)	0.115
Onco‐haematology	4 (3.9)	5 (9.6)	0.155
Others	16 (15.7)	11 (21.2)	0.398
Location of the patient at the time of diagnosis			
ICU	48 (47.1)	39 (75.0)	0.009
Ward	53 (52.9)	13 (25.0)	0.009
Septic shock	28 (27.5)	27 (51.9)	0.003
Type of IAC infection			
Peritonitis	48 (47.1)	24 (46.2)	0.916
Intraabdominal abscess	48 (47.1)	25 (48.1)	0.907
Cholecystitis, cholangitis	5 (4.9)	2 (3.9)	0.779
Infected pancreatic necrosis	1 (0.9)	1 (1.9)	0.952
Site of origin			
Oesophageal/Gastric/Duodenum	21 (20.6)	7 (13.5)	0.282
Jejunum/Ileum	13 (12.8)	6 (11.5)	0.817
Colon	15 (14.7)	5 (9.6)	0.375
Liver/Gallbladder	23 (22.6)	12 (23.1)	0.944
Pancreas	25 (24.5)	20 (38.5)	0.072
Renal/Ureter	5 (4.9)	2 (3.9)	0.779
Parenteral nutrition	42 (41.2)	29 (55.8)	0.085
Active chemotherapy treatment	6 (5.8)	4 (7.7)	0.734
Solid organ transplant immunosuppression	19 (18.6)	9 (17.3)	0.841
Other immunosuppression	3 (2.9)	4 (7.7)	0.227
Surgery in the previous month	77 (75.5)	36 (69.2)	0.406
Perforation	42 (41.2)	24 (46.2)	0.555
Antibiotics within four previous days	76 (74.5)	40 (76.9)	0.743
Antifungals within four previous days	30 (29.4)	23 (44.2)	0.067
*Candida* spp. colonisation	14 (13.7)	17 (32.7)	0.006
Concomitant candidemia	5 (4.9)	14 (26.9)	< 0.001
Breakthrough IAC	5 (4.9)	11 (21.2)	0.002
Type of source control			
Surgical procedure	77 (75.5)	36 (69.2)	0.406
Percutaneous drainage	25 (24.5)	16 (30.8)	0.406
Time from culture obtention to antifungal start, median [IQR] days^b^	2 [0–5]	2 [0–3]	0.567
Hospital length of stay, median [IQR] days	28.5 [17–52]	37 [23–61]	0.042
ICU length of stay, median [IQR] days	6.5 [3–14]	16 [10–28]	< 0.001

Abbreviations: IAC, Intra‐abdominal candidiasis; ICU, Intensive Care Unit; IQR, Interquartile Range; SD, Standard Deviation.

^a^ *p* < 0.05 was considered significant.

^b^ Only included patients without previous antifungal treatment or an active treatment at the beginning of the infection (*N* = 106).

### 3.2 Microbiology

A total of 174 *Candida* spp. were recovered from the 154 patients. Nineteen patients had a mixed fungal infection with two different *Candida* spp., and one patient had three different species. *C. albicans* was most frequently isolated in the azole‐treatment group (49.1% vs. 15.8%, *p* < 0.001), similarly to *C. tropicalis* (15.8% vs. 1.8%, *p* = 0.005) (Table 2). Polymicrobial infections were present in 76.0% of the cases. *Enterococcus faecium* was more frequently isolated in the echinocandin‐treatment group (29.1% vs. 17.9%, *p* = 0.009), while *Streptococcus* spp. in the azole group (19.6% vs. 7.3%, *p* = 0.004).

**TABLE 2:** Isolated *Candida* spp. in the intra‐abdominal candidiasis episodes.

Intraabdominal isolates	*N* total (%)	Azole treatment *N* (%)	Echinocandin treatment *N* (%)	*p* ^a^
**Isolated *Candida* (*N* = 174)**				
> 1 *Candida* spp isolate	20 (13.0)	12 (11.8)	8 (15.4)	0.504
*Candida albicans*	74 (42.5)	56 (49.1)	18 (15.8)	< 0.001
*Candida parapsilosis*	8 (4.6)	5 (4.4)	3 (2.6)	0.555
*Nakaseomyces glabratus* (*Candida glabrata*)	48 (27.6)	19 (16.7)	29 (25.4)	0.172
*Candida tropicalis*	20 (11.5)	18 (15.8)	2 (1.8)	0.005
*Pichia kudriavzevii* ( *Candida krusei* )	13 (7.5)	7 (6.1)	6 (5.3)	0.831
*Candida dubliniensis*	4 (2.3)	3 (2.6)	1 (0.9)	0.449
*Candida lusitaniae*	2 (1.1)	2 (1.8)	0 (0)	0.297
Other *Candida* spp.	5 (2.9)	4 (3.5)	1 (0.9)	0.307

^a^ *p* < 0.05 was considered significant.

Antifungal susceptibility testing was performed on 16.1% (28/174) of the isolates. Among the six *C. albicans* isolates, all were susceptible to azoles, echinocandins, and amphotericin B. Of the four *C. parapsilosis* isolates, 50% exhibited resistance to fluconazole. Regarding the twelve *N. glabratus* isolates, 91.7% (11/12) demonstrated dose‐dependent susceptibility to fluconazole (MIC: 4–16 mg/L), with only one isolate showing resistance (MIC > 32 mg/L). All *N. glabratus* isolates were susceptible to echinocandins. Three of the *C. tropicalis* isolates were susceptible to fluconazole, while one showed dose‐dependent susceptibility (MIC: 4 mg/L).

### 3.3 Antifungal Treatment

A total of 204 antifungal treatments were administered across the 154 analysed episodes of IAC. Based on the antifungal used within the first 5 days following diagnosis, 102 patients (66.2%) received azoles, almost exclusively fluconazole (99.0%, 101/102). The remaining 52 patients were treated with echinocandins, most frequently anidulafungin (90.4%, 47/52), followed by micafungin (5.8%, 3/52) and caspofungin (3.8%, 2/52). Five patients out of seven with *P. kudriavzevii* isolation in the azole group changed to an echinocandin after > 5 days of treatment.

### 3.4 Clinical Outcomes

The outcomes were evaluated for 154 patients who received antifungal treatment for > 3 days. The length of antifungal treatment was shorter in the group primarily treated with azoles than in the group treated with echinocandins (15 [8–25] vs. 20 [13–33.5] days, *p* = 0.014). Among patients who initially underwent surgical intervention, a second procedure was required in 47.7% (54/113), occurring more frequently in the echinocandin‐treated group (77.8% vs. 33.8%, *p* < 0.001). The mortality at the end of treatment was 17.8% for the azole group vs. 34.6% for the echinocandin group (*p* = 0.020); and 30‐day mortality, 22.8% for the azole group vs. 38.5% for the echinocandin group (*p* = 0.041). Ten patients (6.5%) required reinitiation of antifungal therapy for IAC‐related causes within 3 months after treatment completion, despite the absence of positive cultures, with no differences between treatment groups. Microbiological confirmation of recurrence was documented in only one patient (0.6%) (Table 3).

**TABLE 3:** Clinical outcomes of patients with intra‐abdominal candidiasis according to the antifungal treatment within the first five days.

	Total (*N* = 154)	Azole treatment (*N* = 102)	Echinocandin treatment (*N* = 52)	*p* ^a^
Length of antifungal treatment, days, median [IQR]	17 [10–27]	15 [8–25]	20 [13–33.5]	0.014
Persistent positive cultures^b^, *n* (%)	46 (51.7)	26 (51.0)	20 (52.6)	0.877
Need of re‐intervention, *n* (%)^c^	54 (47.8)	26 (33.8)	28 (77.8)	< 0.001
Antifungal reintroduction, *n* (%)	10 (6.5)	8 (7.8)	2 (3.8)	0.341
Mortality end of treatment, *n* (%)	36 (23.5)^d^	18 (17.8)	18 (34.6)	0.020
30‐day mortality, *n* (%)	43 (28.1)^d^	23 (22.8)	30 (38.5)	0.041
90‐day mortality, *n* (%)	47 (30.5)^e^	26 (25.5)	21 (40.4)	0.091

Abbreviation: SD, Standard deviation.

^a^ *p* < 0.05 was considered significant.

^b^ Referred to patients with follow‐up cultures, *n* = 89, (77 in the azole group and 36 in the echinocandin group).

^c^ Referred to patients who initially underwent a surgical intervention, *n* = 113, (51 in the azole group and 38 in the echinocandin group).

^d^ Data missing from one patient.

^e^ Data missing from two patients.

### 3.5 Factors Related to Persistent Positive Cultures

Forty‐six patients (51.7%) out of the 89 cases with follow‐up cultures had persistent positive cultures. The characteristics of patients with or without persistent positive cultures are shown in Table 4. It is of note that the 30‐day mortality rate was significantly higher among those with persistent positive cultures (39.1% vs. 16.7%; *p* = 0.02), as well as the need for re‐intervention after the IAC diagnosis (55.9% vs. 29.6%; *p* = 0.04). Factors associated with persistence were concomitant candidemia (OR: 16.5, 95% CI: 2.0–133.0; *p* = 0.008), septic shock (OR: 4.8, 95% CI: 1.8–12.5; *p* = 0.001), previous use of antibiotics (OR: 3.2, 95% CI: 1.2–9.1; *p* = 0.021), and peritonitis (OR: 4.1, 95% CI: 1.7–10.2; *p* = 0.002) (Table S1).

**TABLE 4:** Clinical characteristics and main outcomes of patients with follow‐up cultures according to the definition of persistent or non‐persistent positive cultures (see methods section).

	Non‐persistent IAC (*N* = 43)	Persistent IAC (*N* = 46)	*p*
Age (years), median [IQR]	61.9 [50.3–74.3]	66.8 [60.0–74.1]	0.144
Male Sex	29 (67.4)	27 (58.7)	0.393
Septic shock	8 (18.6)	24 (52.2)	0.001
Peritonitis	11 (25.6)	27 (58.7)	0.002
Parenteral nutrition	21 (48.8)	27 (58.7)	0.351
Active chemotherapy treatment	2 (4.7)	5 (10.9)	0.436
Solid organ transplant immunosuppression	11 (25.6)	7 (15.2)	0.224
Other immunosuppression	1 (2.3)	1 (2.2)	1.000
Surgery in the previous month	27 (62.8)	34 (73.9)	0.259
Antibiotics within 4 previous days	27 (62.8)	39 (84.8)	0.018
Antifungals within 4 previous days	14 (32.6)	21 (45.7)	0.206
*Candida* spp. colonisation	11 (25.6)	12 (26.1)	0.957
Concomitant candidemia	1 (2.3)	13 (28.3)	0.001
Breakthrough IAC	4 (9.3)	6 (13.0)	0.577
Type of source control			
Surgical procedure	27 (62.8)	34 (73.9)	0.259
Percutaneous drainage	16 (37.2)	12 (26.1)	0.259
Azol treatment	25 (58.1)	26 (56.5)	0.877
Hospital length of stay, median [IQR] days	43 [26–93]	44 [29–76]	0.931
ICU length of stay, median [IQR] days	9 [4–22]	17 [8–28]	0.272
Length of antifungal treatment, days, median [IQR]	21 [15–36]	23.5 [14–34]	0.902
Need of re‐intervention, *n* (%)^b^	8 (29.6)	19 (55.9)	0.040
Mortality end of treatment, *n* (%)	5 (11.9)	16 (34.8)	0.012
30‐day mortality, *n* (%)	7 (16.7)	18 (39.1)	0.020

Abbreviations: IAC, Intra‐abdominal candidiasis; IQR, Interquartile Range.

^a^ *p* < 0.05 was considered significant.

^b^ Referred to patients who initially underwent a surgical intervention, *n* = 61 (27 in the non‐persistent group and 34 in the persistent group).

### 3.6 Factors Related to 30‐Day Mortality

**FIGURE 1:**
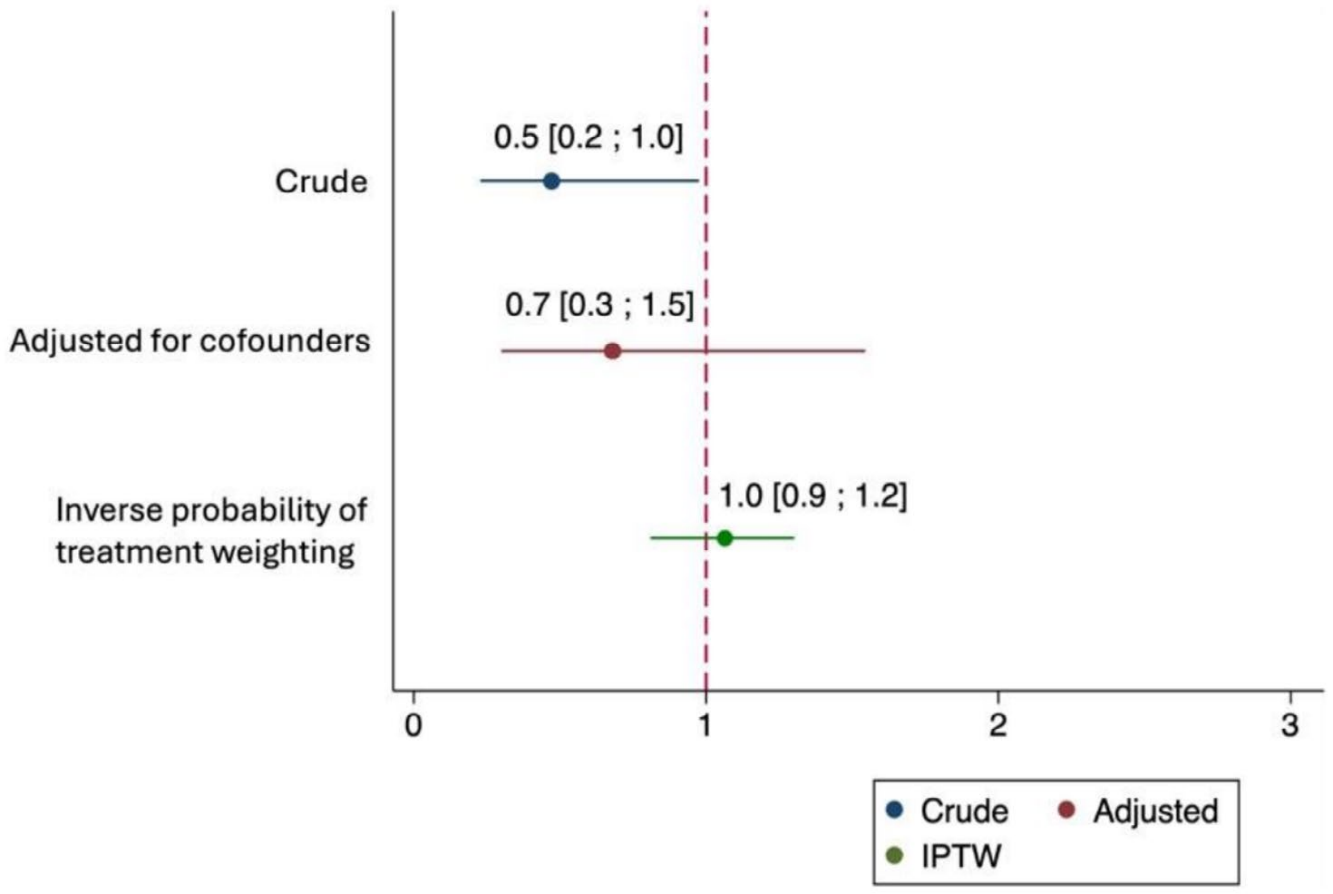
Estimates of initial antifungal treatment on 30‐day mortality. CI, Confidence Interval; IPTW, Inverse Probability of Treatment Weighting.

A total of 43 patients (28.1%) died within 30 days of ending antifungal treatment. Crude 30‐day mortality was lower among patients treated with azoles than among those receiving echinocandins (OR 0.5, 95% CI 0.2–1.0; *p* = 0.043). However, after adjustment for confounders in the multivariate regression there was no association between antifungals and 30‐day mortality (OR: 0.7, 95% CI: 0.3–1.5; *p* = 0.357) (Figure 1). The multivariate analysis showed that the presence of septic shock (OR: 2.2, 95% CI: 1.0–4.9; *p* = 0.047) and age ≥ 60 years (OR: 2.6, 95% CI: 1.1–6.3; *p* = 0.032) were significantly associated with 30‐day mortality. Concomitant candidemia showed a trend towards a higher mortality, although it was not statistically significant (OR: 2.8, 95% CI: 0.9–8.6; *p* = 0.08) (Table S2). To account for non‐randomised treatment allocation, we applied IPTW (Table S3). The estimated ATET showed no difference in 30‐day mortality between azoles and echinocandins (coefficient 0.04; 95% CI –0.1–0.2; *p* = 0.563).

## 4 Discussion

This single‐center study offers valuable insights into the management of IAC, comparing clinical outcomes based on the initial antifungal therapy. Patients receiving azoles exhibited more favourable outcomes in terms of both end‐of‐treatment and 30‐day mortality; however, this group had a lower proportion of cases with septic shock and concomitant candidemia, which may have contributed to the observed differences. Indeed, IPTW and multivariate logistic regression analyses did not identify antifungal therapy as a variable associated with either 30‐day mortality or persistent positive cultures. Nevertheless, despite source control, both the rate of persistent positive cultures (51.7%) and 30‐day mortality (28.1%) remained high in both groups, likely reflecting in part the baseline severity and comorbidities of these patients.

Mortality was independent of the initial antifungal therapy, although patients primarily treated with echinocandins exhibited poorer outcomes, which may be attributed to four main factors. First, the echinocandins were selected for more severe patients with a higher proportion of patients with septic shock (51.9% vs. 27.5%, *p* = 0.002). Second, recent studies have shown that standard echinocandin dosing is not enough to achieve the PK‐PD target in critically ill patients with increased body weight, hypoalbuminemia, septic shock, or increased MIC to echinocandins [21, 22, 23]. Third, the suboptimal penetration of echinocandins into the peritoneal cavity may limit their efficacy [14, 18, 19, 24, 25, 26]. Fourth, this group more often exhibited co‐infection with *E. faecium* , a nosocomial pathogen with poor response to antibiotic treatment [27], and had a higher proportion of pancreatic abscesses (70% vs. 45.8%), a condition that typically shows poor response to antifungal therapy [26].

Liposomal amphotericin B has potent concentration‐dependent fungicidal activity against *Candida* spp., with a low risk of resistance [2, 17, 28]. However, clinical data supporting its use in IAC is scarce, and the safety profile is always concerning [17].

In our cohort, the overall 30‐day mortality was 28.1%, consistent with previous studies reporting mortality rates between 18%–27% in general hospital settings and 40%–52% in ICU patients [5, 12, 13, 29], although mortality differed between patients with peritonitis (32.4%) and those with abscesses (24.7%). Similar to our findings, Vergidis et al. [5] identified age, abscess vs. peritonitis, and early source control as independent predictors of survival in IAC. Age‐related immune dysfunction, characterised by altered cytokine responses and reduced monocyte and neutrophil activity, may predispose elderly patients to more severe outcomes [30]. In critically ill populations, Dupont et al. [13] reported that APACHE II scores > 17, respiratory failure, upper gastrointestinal peritonitis, and *Candida* isolation were independently associated with mortality. These findings were corroborated by Bassetti et al. [31], who also identified severe clinical presentation and candidemia as mortality risk factors, results that align with our observations.

Data on the rate of persistent positive cultures in IAC is scarce; we observed a frequency of 51.7%, irrespective of antifungal therapy. This finding was primarily associated with candidemia, which also showed a trend towards increased 30‐day mortality (*p* = 0.08), as well as with septic shock, peritonitis, and prior antibiotic use. In fact, the 30‐day mortality was significantly higher in patients with persistent positive cultures than in those without (39.1% vs. 16.7%; *p* = 0.02). Obtaining cultures through intra‐abdominal catheters remains controversial, as many authors consider these samples to be inherently prone to contamination [32]. However, our findings indicate that persistent positivity of cultures likely results from infections with a high initial fungal load and insufficient source control. Therefore, we consider this practice useful for monitoring the patients' evolution and as an early marker of the need for re‐intervention that finally was more common in this group (Table 4).

The present study enhances our understanding of IAC by highlighting the current challenges in diagnosis and treatment, as well as the significance of persistent positive cultures from intra‐abdominal samples. Although echinocandins and fluconazole have been commonly used and associated with comparable clinical response, overall outcomes remain variable, and mortality rates are still high. Azoles exhibit superior tissue penetration, although recommending them as first‐line therapy is controversial, as in our cohort, more than one third of *Candida* isolates belonged to species with absent or variable susceptibility to fluconazole (*P. kudriavzevii*, *N. glabratus* , and *C. parapsilosis* ). However, once species identification and susceptibility data are available, de‐escalation to fluconazole is a reasonable option given its better penetration and higher likelihood of target attainment [26].

However, several limitations should be acknowledged. First, the retrospective non‐randomised design and single‐center setting may limit the generalizability of the results due to potential regional differences in *Candida* epidemiology, clinical practices, and healthcare systems. Second, in the diagnosis of most IAC cases, biomarkers such as β‐D‐glucan (BDG) were not available despite their recognised diagnostic utility [33]. Third, although various risk factors for IAC and underlying comorbidities have been described in the literature, we were unable to collect data on some relevant variables (e.g., underlying comorbidities), which may have influenced outcomes. Finally, the absence of antifungal susceptibility testing in a significant proportion of isolates limits the analysis of antifungal effectiveness, particularly in the case of azoles.

Our findings underscore the difficulties in managing IAC and emphasise the need for the development of new antifungals that not only exhibit potent activity against *Candida* spp. but also achieve high penetration in the abdominal cavity. Future research should include prospective trials to explore the role of those novel antifungal agents and assess the clinical utility and kinetics of non‐culture‐based biomarkers, including BDG in serum and peritoneal fluid.

In conclusion, IAC is still associated with high mortality, particularly among older patients and those presenting with septic shock. A considerable proportion of patients exhibited persistent positive cultures regardless of the antifungal used, especially in cases with concomitant candidemia. Initial antifungal choice did not appear to drive clinical outcomes. These findings underscore the need for improved diagnostic strategies and further research to refine the therapeutic management of IAC.

## Author Contributions

**M. Albanell‐Fernández:** writing – original draft, conceptualization, writing – review and editing, methodology, formal analysis. **A. Vergara:** writing – review, and editing. **F. Marco:** writing – review, and editing. **S. Herrera:** writing – review, and editing. **M. Tuset:** writing – review, and editing. **A. Soriano:** conceptualization, writing – review and editing, methodology, formal analysis, and supervision. **M. Bodro:** conceptualization, writing – review and editing, methodology, formal analysis, and supervision.

## Funding

The authors have nothing to report.

## Conflicts of Interest

The authors declare no conflicts of interest.

## Supporting information


**Table S1:** Predictors of persistent positive *Candida* cultures in patients with follow‐up cultures receiving antifungal treatment for more than 3 days.
**Table S2:** Predictors of 30‐day mortality in subjects with IAC receiving antifungal treatment for more than 3 days.
**Table S3:** Covariate Balance Before and After Inverse Probability of Treatment Weighting (IPTW).

## Data Availability

The data that support the findings of this study are available from the corresponding author upon reasonable request.

## References

[1] M. Bassetti , E. Azoulay , B.‐J. Kullberg , et al., “EORTC/MSGERC Definitions of Invasive Fungal Diseases: Summary of Activities of the Intensive Care Unit Working Group,” Clinical Infectious Diseases 72 (2021): S121–S127, 10.1093/cid/ciaa1751. 33709127

[2] E. Maseda , I. Martín‐Loeches , R. Zaragoza , et al., “Critical Appraisal Beyond Clinical Guidelines for Intraabdominal Candidiasis,” Critical Care 27 (2023): 382, 10.1186/s13054-023-04673-6. 37789338 PMC10546659

[3] K. Habighorst , J. M. Sanders , S. A. Hennessy , K. Goff , B. Wan , and M. Johns , “Identification of Risk Factors for Intra‐Abdominal Candidiasis,” Surgical Infections 24 (2023): 910–915, 10.1089/sur.2023.149. 38011638

[4] M. Bassetti , M. Marchetti , A. Chakrabarti , et al., “A Research Agenda on the Management of Intra‐Abdominal Candidiasis: Results From a Consensus of Multinational Experts,” Intensive Care Medicine 39 (2013): 2092–2106, 10.1007/s00134-013-3109-3. 24105327

[5] P. Vergidis , C. J. Clancy , R. K. Shields , et al., “Intra‐Abdominal Candidiasis: The Importance of Early Source Control and Antifungal Treatment,” PLoS One 11 (2016): e0153247, 10.1371/journal.pone.0153247. 27123857 PMC4849645

[6] M. Bassetti , A. Vena , D. R. Giacobbe , et al., “Risk Factors for Intra‐Abdominal Candidiasis in Intensive Care Units: Results From EUCANDICU Study,” Infectious Disease and Therapy 11 (2022): 827–840, 10.1007/s40121-021-00585-6. PMC8960530 35182353

[7] P. Montravers , H. Dupont , and P. Eggimann , “Intra‐Abdominal Candidiasis: The Guidelines–Forgotten Non‐Candidemic Invasive Candidiasis,” Intensive Care Medicine 39 (2013): 2226–2230, 10.1007/s00134-013-3134-2. 24154676

[8] M. Bassetti , D. R. Giacobbe , A. Vena , et al., “Incidence and Outcome of Invasive Candidiasis in Intensive Care Units (ICUs) in Europe: Results of the EUCANDICU Project,” Critical Care 23 (2019): 219, 10.1186/s13054-019-2497-3. 31200780 PMC6567430

[9] M. Bassetti , M. Peghin , A. Carnelutti , et al., “Invasive Candida Infections in Liver Transplant Recipients: Clinical Features and Risk Factors for Mortality,” Transplantation Direct 3 (2017): e156, 10.1097/TXD.0000000000000673. 28573191 PMC5441987

[10] B. L. de Almeida , V. C. Arcieri , D. M. Razente , et al., “Intra‐Abdominal Candidiasis in Cancer Patients: A 10‐Year Experience in a Middle‐Income Country,” Mycoses 67 (2024): e13807, 10.1111/myc.13807. 39455432

[11] T. Yan , S. Li , H. Ou , S. Zhu , L. Huang , and D. Wang , “Appropriate Source Control and Antifungal Therapy Are Associated With Improved Survival in Critically Ill Surgical Patients With Intra‐Abdominal Candidiasis,” World Journal of Surgery 44 (2020): 1459–1469, 10.1007/s00268-020-05380-x. 31965275

[12] M. Bassetti , E. Righi , F. Ansaldi , et al., “A Multicenter Multinational Study of Abdominal Candidiasis: Epidemiology, Outcomes and Predictors of Mortality,” Intensive Care Medicine 41 (2015): 1601–1610, 10.1007/s00134-015-3866-2. 26077063

[13] H. Dupont , C. Paugam‐Burtz , C. Muller‐Serieys , et al., “Predictive Factors of Mortality due to Polymicrobial Peritonitis With Candida Isolation in Peritoneal Fluid in Critically Ill Patients,” Archives of Surgery 137 (2002): 1341–1346, 10.1001/archsurg.137.12.1341. 12470095

[14] M. M. Pais , R. Zaragoza , I. Martín‐Loeches , F. F. Gómez‐Bertomeu , and A. Rodríguez , “Management of Intra‐Abdominal Candidiasis in Intensive Care Setting: A Narrative Review,” Journal of Fungi 11 (2025): 362, 10.3390/jof11050362. 40422696 PMC12112819

[15] J. S. Solomkin , J. E. Mazuski , J. S. Bradley , et al., “Diagnosis and Management of Complicated Intra‐Abdominal Infection in Adults and Children: Guidelines by the Surgical Infection Society and the Infectious Diseases Society of America,” Clinical Infectious Diseases 50 (2010): 133–164, 10.1086/649554. 20034345

[16] P. G. Pappas , C. A. Kauffman , D. R. Andes , et al., “Clinical Practice Guideline for the Management of Candidiasis: 2016 Update by the Infectious Diseases Society of America,” Clinical Infectious Diseases 62 (2015): e1–e50, 10.1093/cid/civ933. 26679628 PMC4725385

[17] O. A. Cornely , R. Sprute , M. Bassetti , et al., “Global Guideline for the Diagnosis and Management of Candidiasis: An Initiative of the ECMM in Cooperation With ISHAM and ASM,” Lancet Infectious Diseases 25 (2025): e280–e293, 10.1016/S1473-3099(24)00749-7. 39956121

[18] N. Garbez , L. C. Mbatchi , S. C. Wallis , et al., “Caspofungin Population Pharmacokinetic Analysis in Plasma and Peritoneal Fluid in Septic Patients With Intra‐Abdominal Infections: A Prospective Cohort Study,” Clinical Pharmacokinetics 61 (2022): 673–686, 10.1007/s40262-021-01062-6. 34931282

[19] D. V. Pérez Civantos , M. Robles Marcos , J. R. Azanza Perea , C. Pazos Pacheco , F. García‐Montoto Pérez , and V. Jerez Gómez‐Coronado , “Pharmacokinetics of Anidulafungin in Critically Ill Patients With Candida Peritonitis,” International Journal of Infectious Diseases 86 (2019): 142–146, 10.1016/j.ijid.2019.07.008. 31330325

[20] F. Gioia , A. Gomez‐Lopez , M. E. Alvarez , et al., “Pharmacokinetics of Echinocandins in Suspected Candida Peritonitis: A Potential Risk for Resistance,” International Journal of Infectious Diseases 101 (2020): 24–28, 10.1016/j.ijid.2020.09.019. 32937195

[21] J. A. Roberts , F. B. Sime , J. Lipman , et al., “Are Contemporary Antifungal Doses Sufficient for Critically Ill Patients? Outcomes From an International, Multicenter Pharmacokinetics Study for Screening Antifungal Exposure in Intensive Care Units‐The SAFE‐ICU Study,” Intensive Care Medicine 51 (2025): 302–317, 10.1007/s00134-025-07793-5. 39899034 PMC11903579

[22] O. Elkayal , Y. Hoffert , B. Mertens , et al., “Anidulafungin Exposure and Population Pharmacokinetics in Critically Ill Patients With Invasive Candidiasis,” Infection 53 (2024): 1155–1165, 10.1007/s15010-024-02448-x. 39641856

[23] D. F. Bavaro , L. Diella , A. De Angelis , et al., “Why Do Echinocandins Fail? Identifying Key Predictors to Improve Clinical Outcomes of Candida Bloodstream Infections: A Retrospective Multicentre Cohort Study,” International Journal of Infectious Diseases 160 (2025): 108046, 10.1016/j.ijid.2025.108046. 40912531

[24] N. Garbez , L. Mbatchi , S. C. Wallis , et al., “Prospective Cohort Study of Micafungin Population Pharmacokinetic Analysis in Plasma and Peritoneal Fluid in Septic Patients With Intra‐Abdominal Infections,” Antimicrobial Agents and Chemotherapy 65 (2021): e0230720, 10.1128/AAC.02307-20. 33846133 PMC8218641

[25] M. Albanell‐Fernández , “Echinocandins Pharmacokinetics: A Comprehensive Review of Micafungin, Caspofungin, Anidulafungin, and Rezafungin Population Pharmacokinetic Models and Dose Optimization in Special Populations,” Clinical Pharmacokinetics 64 (2025): 27–52, 10.1007/s40262-024-01461-5. 39707078 PMC11762474

[26] A. Cancela Costa , F. Grass , I. Andres Cano , et al., “Antibacterial and Antifungal Drug Concentrations in Intra‐Abdominal Abscesses: A Prospective Clinical Study,” Antimicrobial Agents and Chemotherapy 69 (2025): e01178‐24, 10.1128/aac.01178-24. 39636126 PMC11784227

[27] M. Rinaldi , I. Rancan , F. Malerba , et al., “ *Enterococcus faecium* Bacteraemia: A Multicentre Observational Study Focused on Risk Factors for Clinical and Microbiological Outcomes,” Journal of Antimicrobial Chemotherapy 80 (2025): 2247–2256, 10.1093/jac/dkaf197. 40577612 PMC12313462

[28] J. Maertens , L. Pagano , E. Azoulay , and A. Warris , “Liposomal Amphotericin B—The Present,” Journal of Antimicrobial Chemotherapy 77 (2022): ii11–ii20, 10.1093/jac/dkac352. 36426672 PMC9693760

[29] G. Sganga , M. Wang , M. R. Capparella , et al., “Evaluation of Anidulafungin in the Treatment of Intra‐Abdominal Candidiasis: A Pooled Analysis of Patient‐Level Data From 5 Prospective Studies,” European Journal of Clinical Microbiology & Infectious Diseases 38 (2019): 1849–1856, 10.1007/s10096-019-03617-9. 31280481 PMC6778589

[30] J. Lu , J. Liu , L. Zhu , Y. Zhang , and A. Li , “The Effect of Age on the Clinical Characteristics and Innate Immune Cell Function in the Patients With Abdominal Sepsis,” Frontiers in Physiology 13 (2022): 13, 10.3389/fphys.2022.952434. PMC9551265 36237524

[31] M. Bassetti , M. Peghin , A. Carnelutti , et al., “Clinical Characteristics and Predictors of Mortality in Cirrhotic Patients With Candidemia and Intra‐Abdominal Candidiasis: A Multicenter Study,” Intensive Care Medicine 43 (2017): 509–518, 10.1007/s00134-017-4717-0. 28271321

[32] R. J. Everts , J. P. Heneghan , P. O. Adholla , and L. B. Reller , “Validity of Cultures of Fluid Collected Through Drainage Catheters Versus Those Obtained by Direct Aspiration,” Journal of Clinical Microbiology 39 (2001): 66–68, 10.1128/JCM.39.1.66-68.2001. 11136750 PMC87681

[33] C. Deckers , I. Montesinos , P. E. Plum , M. Bassetti , and P. M. Honoré , “Invasive *Candida* in the Abdomen: How to Differentiate Infection From Colonization,” Expert Review of Anti‐Infective Therapy 23 (2025): 585–595, 10.1080/14787210.2025.251655. 40492348

