# Supplementary material for: Clinical Outcomes of Intra‐Abdominal Candidiasis by Initial Antifungal Therapy
Source: Mycoses. 2026 Mar 3;69(3):e70147. doi: 10.1111/myc.70147 (PMC12955698; doi:10.1111/myc.70147)
Supplement: Supplementary file 1 — Table S1: Predictors of persistent positive Candida cultures in patients with follow‐up cultures receiving antifungal treatment for more than 3 days. Table S2: Predictors of 30‐day mortality in subjects with IAC receiving antifungal treatment for more than 3 days. Table S3: Covariate Balance Before and After Inverse Probability of Treatment Weighting (IPTW). [file MYC-69-e70147-s001.docx]

**Table S1.** Predictors of persistent positive *Candida* cultures in patients with follow-up cultures receiving antifungal treatment for more than three days.

| **Parameter** | **Non-persistent IAC (N = 43)** | **Persistent IAC (N = 46)** | **Univariate OR**  **(95% CI)** | ***P-value***^a^ |
| --- | --- | --- | --- | --- |
| Age (≥60 years) | 25 (58.1) | 35 (76.1) | 2.3 (1.0 – 5.7) | 0.074 |
| Septic shock | 8 (18.6) | 24 (52.2) | 4.8 (1.8 – 12.5) | 0.001 |
| Peritonitis | 11 (25.6) | 27 (58.7) | 4.1 (1.7 – 10.2) | 0.002 |
| Antibiotics within 4 previous days | 27 (62.8) | 39 (84.8) | 3.2 (1.2 – 9.1) | 0.021 |
| Candidemia | 1 (2.3) | 13 (28.3) | 16.5 (2.0 – 133.0) | 0.008 |

Abbreviations: CI: Confidence interval; OR: Odds ratio.

^a^Statistical significance tested by Pearson's chi-squared. A *P-value* of <0.05 was considered significant.

**Table S2.** Predictors of 30-day mortality in subjects with IAC receiving antifungal treatment for more than three days.

| **Parameter** | **30-day survivors (N = 110)^a^** | **30-day non-survivors (N = 43)^a^** | **Univariate OR**  **(95% CI)** | ***P-value***^b^ | **Multivariate OR**  **(95% CI)** | ***P-value***^b^ |
| --- | --- | --- | --- | --- | --- | --- |
| Age (≥60 years) | 64 (58.2) | 34 (79.1) | 2.7 (1.2 – 6.2) | 0.018 | 2.6 (1.1 – 6.3) | 0.032 |
| Septic shock | 32 (29.1) | 23 (53.5) | 2.8 (1.4 – 5.8) | 0.005 | 2.2 (1.0 – 4.9) | 0.047 |
| Candidemia | 9 (8.2) | 10 (23.3) | 3.4 (1.3 – 9.1) | 0.015 | 2.8 (0.9 – 8.6) | 0.080 |
| Azol-treatment | 78 (70.9) | 23 (53.5) | 0.5 (0.2 – 1.0) | 0.043 | 0.7 (0.3 – 1.5) | 0.357 |
| Persistence | 28 (25.5) | 18 (41.9) | 2.1 (1.0 – 4.4) | 0.049 | 1.2 (0.5 – 2.8) | 0.695 |

Abbreviations: CI: Confidence interval; OR: Odds ratio.

^a^Data missing from one patient (N=153).

^b^Statistical significance tested by Pearson's chi-squared. A *P-value* of <0.05 was considered significant.

**Table S3.** Covariate Balance Before and After Inverse Probability of Treatment Weighting (IPTW).

| **Covariate** | **SMD (Raw)** | **SMD (Weighted)** | **Variance Ratio (Raw)** | **Variance Ratio (Weighted)** |
| --- | --- | --- | --- | --- |
| Male Sex | 0.0408 | –0.0152 | 0.963 | 1.012 |
| Age (≥60 years) | 0.0185 | –0.0338 | 0.980 | 1.022 |
| Septic shock | –0.5063 | –0.1086 | 0.795 | 0.910 |
| Presence of peritonitis | 0.0076 | –0.1107 | 0.992 | 0.997 |
| Parenteral nutrition | –0.2846 | –0.1191 | 0.976 | 0.974 |
| Active chemotherapy treatment | –0.0690 | 0.0125 | 0.780 | 1.049 |
| Solid organ transplant immunosuppression | 0.0388 | –0.0786 | 1.057 | 0.890 |
| Other immunosuppression | –0.2096 | 0.0224 | 0.402 | 1.138 |
| Surgery in the previous month | 0.1337 | –0.1413 | 0.866 | 1.216 |
| Antibiotics within 4 previous days | –0.0390 | –0.1190 | 1.039 | 1.174 |
| Antifungals within 4 previous days | –0.3021 | –0.0350 | 0.839 | 0.971 |
| *Candida* spp. colonization | –0.4534 | 0.0698 | 0.538 | 1.171 |
| Concomitant candidemia | –0.6238 | 0.0567 | 0.237 | 1.293 |

Abbreviation: Standardized Mean Differences
